# Faecal microRNAs as a non-invasive tool in the diagnosis of colonic adenomas and colorectal cancer: A meta-analysis

**DOI:** 10.1038/s41598-019-45570-9

**Published:** 2019-07-01

**Authors:** Tung On Yau, Ceen-Ming Tang, Elinor K. Harriss, Benjamin Dickins, Christos Polytarchou

**Affiliations:** 1 Department of Biosciences, John van Geest Cancer Research Centre, School of Science and Technology, Nottingham Trent University, Nottingham, UK; 2 Oxford University Clinical Academic Graduate School, John Radcliffe Hospital, Oxford, UK; 3 Bodleian Health Care Libraries, University of Oxford, Oxford, UK

**Keywords:** Colorectal cancer, Diagnostic markers

## Abstract

MicroRNAs (miRNAs) are proposed as potential biomarkers for the diagnosis of numerous diseases. Here, we performed a meta-analysis to evaluate the utility of faecal miRNAs as a non-invasive tool in colorectal cancer (CRC) screening. A systematic literature search, according to predetermined criteria, in five databases identified 17 research articles including 6475, 783 and 5569 faecal-based miRNA tests in CRC, adenoma patients and healthy individuals, respectively. Sensitivity, specificity, positive/negative likelihood and diagnostic odds ratios, area under curve (AUC), summary receiver operator characteristic (sROC) curves, association of individual or combinations of miRNAs to cancer stage and location, subgroup, meta-regression and Deeks’ funnel plot asymmetry analyses were employed. Pooled miRNAs for CRC had an AUC of 0.811, with a sensitivity of 58.8% (95% confidence interval [CI]: 51.7–65.5%) and specificity of 84.8% (95% CI: 81.1–87.8%), whilst for colonic adenoma, it was 0.747, 57.3% (95% CI: 40.8–72.3%) and 76.1% (95% CI: 66.1–89.4%), respectively. The most reliable individual miRNA was miR-21, with an AUC of 0.843, sensitivity of 59.3% (95% CI: 26.3–85.6%) and specificity of 85.6% (95% CI: 72.2–93.2%). Paired stage analysis showed a better diagnostic accuracy in late stage CRC and sensitivity higher in distal than proximal CRC. In conclusion, faecal miR-21, miR-92a and their combination are promising non-invasive biomarkers for faecal-based CRC screening.

## Introduction

Colorectal cancer (CRC) is the second leading cancer-related cause of death in the United Kingdom (UK) and accounts for over 500,000 deaths annually worldwide^1^. The pathogenesis of CRC follows a protracted stepwise progression from benign colonic adenomas to malignant adenocarcinomas and distant metastasis. Patient survival inversely correlates to cancer stage during diagnosis, with up to 90% of deaths avertable if detected early^2^. However, CRC is often asymptomatic in its early stage and arises sporadically within the population, posing a challenge to the application of effective and timely treatments^3^. The mass screening of asymptomatic individuals for CRC utilising a non-invasive method is thus a high public health priority.

Under the National Health Service (NHS) Bowel Cancer Screening Program in the UK, currently, the faecal immunochemical test (FIT) is offered every two years to all asymptomatic men and women aged 60 to 74^4^. The FIT, which examines faecal samples for hidden blood, is appealing because the costs are low, the test is widely available, and does not pose an immediate risk to the screened population^5^. Although the recent changes from Faecal Occult Blood Test (FOBT) to FIT has improved the screening power by specific targeting to human haemoglobin, the effectiveness of FIT is still restricted by its relatively low sensitivity, with about half of all malignant large bowel tumours and most polyps undetected. This is due to the intermittent nature of bleeding^6^ as well as degradation of haemoglobin in faeces^7^. Consequently, one in four CRC cases is only diagnosed at a late stage on emergency admission, resulting in poor prognosis^8^. Therefore, a more sensitive faecal-based non-invasive test is urgently needed.

miRNAs are a class of conserved endogenous, short non-coding RNAs with length of 18–24 nucleotides. miRNAs regulate gene expression through post-transcriptional processing by binding primarily to the 3′-untranslated region (3′UTR) of target mRNAs, resulting in mRNA degradation and/or translational repression^9^. Specific miRNAs (oncomiRs) through targeting tumour-suppressor genes have been found to be upregulated, while others targeting oncogenes are downregulated, in cancer. These alterations, through the regulation of intracellular signalling networks, induce cell proliferation, confer resistance to apoptosis and chemotherapy, and promote metastasis^10^. The expression of several miRNAs differs significantly between normal colonic tissues and CRC, and as colonocytes consistently exfoliate and shed into the lumen of the gastrointestinal (GI) tract, these changes in miRNA levels are represented in faecal specimens^11–27^. More recently, it was demonstrated that miRNAs are highly stable and detectable within samples throughout a 72 hour incubation period due to protection from ribonuclease degradation by exosomes^28,29^. Given that faeces contain genomic DNA and RNA derived from gut microbes, and miRNAs derived from blood cells released by tumours, the detection and utility of miRNAs for diagnostic purposes has been controversial. Therefore, this meta-analysis aims to assess the value of miRNAs as faecal-based biomarkers for CRC and colonic adenoma screening.

## Results

### Characteristics of selected studies

**Figure 1:**
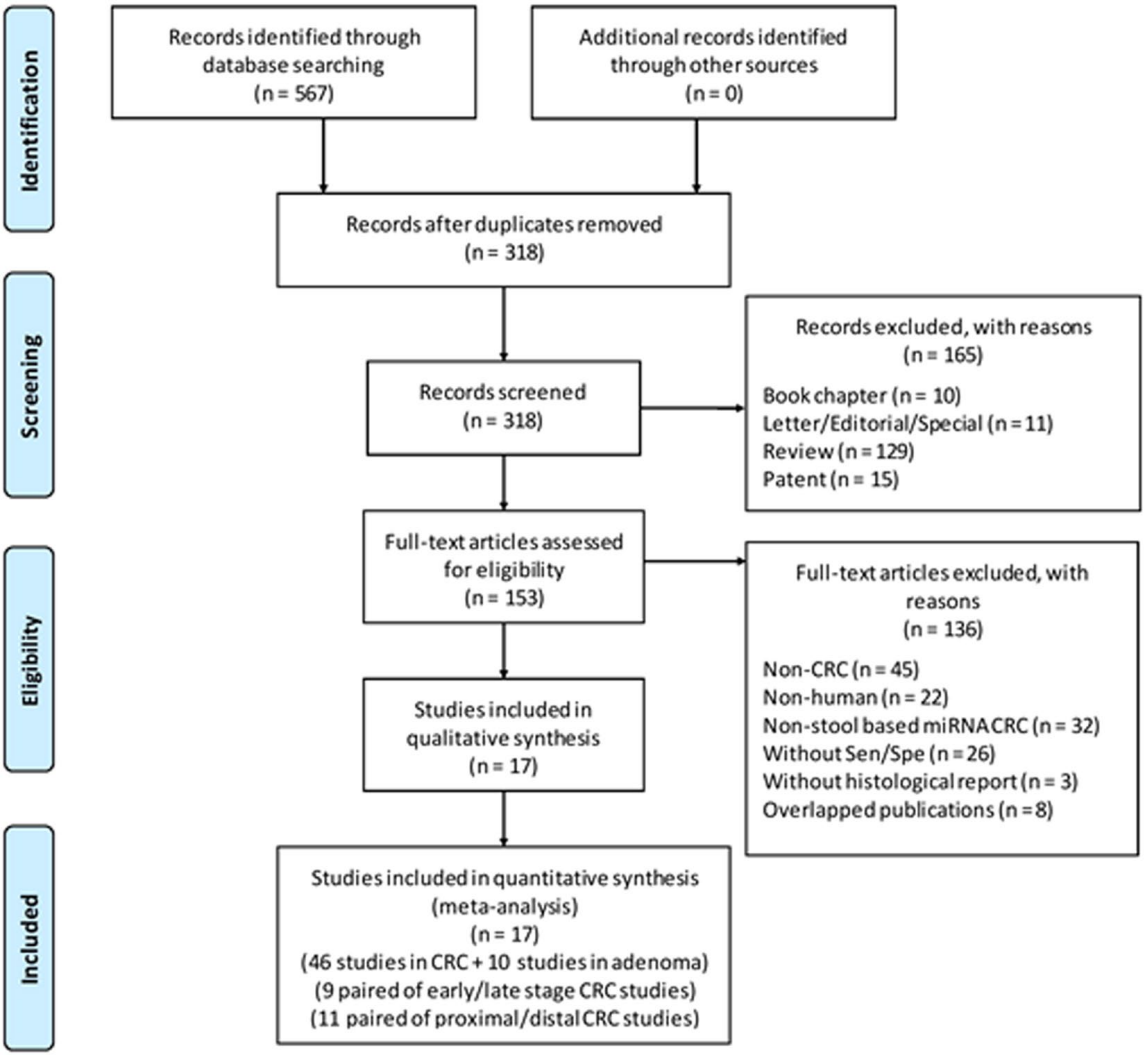
Flowchart diagram of study selection based on the inclusion and exclusion criteria.

The initial literature search from five different databases yielded a total of 567 articles, of which 249 were excluded as duplicated records. Next, 165 articles were deemed irrelevant and excluded based on the title and abstract. The full-text of the remaining publications were screened, resulting in the inclusion of 17 publications (16 in English, 1 in Chinese) (Fig. 1). These publications contained 46 studies on CRC and 10 studies on adenomas, corresponding to 6475, 783 and 5569 faecal-based miRNA tests in CRC patients, adenoma and healthy controls, respectively (Tables 1 and 2). The clinical data and collection procedures are summarised in Suppl. Table 1A,B, and methods of miRNA extraction and quantification in Suppl. Table 2A,B.

**Table 1:** All publications on faecal-based microRNAs for the detection of colorectal cancer.

Study ID	First author (Year)[Reference no.]	Origin of population	Sample size		miRNA profile	qPCR quantitation method	Proximal/Distal	Early/Late stage
			CRC	Control				
1	Koga Y (2010)^11^	Japan	119	197	miR-17-92 cluster*	Relative	Y	Y
2	Koga Y (2010)^11^	Japan	119	197	miR-17	Relative	—	—
3	Koga Y (2010)^11^	Japan	119	197	miR-18a	Relative	—	—
4	Koga Y (2010)^11^	Japan	119	197	miR-19a	Relative	—	—
5	Koga Y (2010)^11^	Japan	119	197	miR-19b	Relative	—	—
6	Koga Y (2010)^11^	Japan	119	197	miR-20a	Relative	—	—
7	Koga Y (2010)^11^	Japan	119	197	miR-92a	Relative	—	—
8	Koga Y (2010)^11^	Japan	119	197	miR-21	Relative	Y	Y
9	Koga Y (2010)^11^	Japan	119	197	miR-135a, miR-135b	Relative	Y	Y
10	Koga Y (2010)^11^	Japan	119	197	miR-135a	Relative	—	—
11	Koga Y (2010)^11^	Japan	119	197	miR-135b	Relative	—	—
12	Koga Y (2010)^11^	Japan	119	197	miR-17-92 cluster*, miR-21, miR-135a/b	Relative	Y	Y
13	Kalimutho M (2011)^12^	Italy	40	35	miR-144-5p	Relative	—	—
14	Wu CW (2012)^20^	Hong Kong	101	88	miR-21	Absolute	Y	Y
15	Wu CW (2012)^20^	Hong Kong	101	88	miR-92a	Absolute	Y	Y
16	Wu CW (2012)^20^	Hong Kong	101	88	miR-21, miR-92a	Absolute	Y	Y
17	Kuriyama S (2012)^21^	Japan	126	138	miR-106a	Relative	—	—
18	Kuriyama S (2012)^21^	Japan	126	138	miR-21, miR-92a, miR-106a	Relative	—	—
19	Kanaoka S (2013)^22^	Japan	126	138	miR-21	Relative	—	—
20	Kanaoka S (2013)^22^	Japan	126	138	miR-92a	Relative	—	—
21	Koga Y (2013)^23^	Japan	107	117	miR-106a	Relative	—	—
22	Zhao HJ (2014)^24^	China	20	28	miR-194	Relative	—	—
23	Wu CW (2014)^25^	Hong Kong	109	104	miR-135b	Absolute	—	—
24	Yau TO (2014)^26^	Hong Kong	198	198	miR-221	Absolute	—	—
25	Yau TO (2014)^26^	Hong Kong	198	198	miR-18a	Absolute	—	—
26	Yau TO (2014)^26^	Hong Kong	198	198	miR-221, miR-18a	Absolute	—	—
27	Yau TO (2014)^26^	Hong Kong	198	198	miR-221, miR-135b	Absolute	—	—
28	Yau TO (2014)^26^	Hong Kong	198	198	miR-18a, miR-135b	Absolute	—	—
29	Yau TO (2014)^26^	Hong Kong	198	198	miR-221, miR-18a, miR-135b	Absolute	—	—
30	Phua LC (2014)^27^	Singapore	28	17	miR-223	Relative	—	—
31	Phua LC (2014)^27^	Singapore	28	17	miR-451	Relative	—	—
32	Chang PY (2016)^13^	Taiwan	309	138	miR-223, miR-92a, miR-16, miR-106b	Relative	—	—
33	Chang PY (2016)^13^	Taiwan	309	138	miR-223, miR-92a	Relative	—	—
34	Yau TO (2016)^14^	Hong Kong	198	198	miR-20a	Absolute	—	—
35	Yau TO (2016)^14^	Hong Kong	198	198	miR-20a, miR-92a	Absolute	—	—
36	Yau TO (2016)^14^	Hong Kong	198	198	miR-20a, miR-135b	Absolute	—	—
37	Zhu Y (2016)^15^	China	51	80	miR-29a	Relative	—	—
38	Zhu Y (2016)^15^	China	51	80	miR-223	Relative	—	—
39	Zhu Y (2016)^15^	China	51	80	miR-224	Relative	—	—
40	Liu H (2016)^16^	China	150	98	miR-21	Relative	—	—
41	Liu H (2016)^16^	China	150	98	miR-146a	Relative	—	—
42	Liu H (2016)^16^	China	150	98	miR-21, miR-146a	Relative	—	—
43	Xue Y (2016)^17^	China	50	50	miR-141	Relative	—	—
44	Xue Y (2016)^17^	China	50	50	miR-92a	Relative	—	—
45	Bastaminejad S (2017)^18^	Iran	40	40	miR-21	Relative	—	—
46	Wu CW (2017)^19^	USA	29	115	miR-451a, miR-144-5p	Relative	Y	Y

^*^The miR-17-92 cluster includes miR-17, miR-18a, miR-19a, miR-20a, miR-19b-1, and miR-92a.

**Table 2:** All publications on faecal-based microRNAs for the detection of colonic adenomas.

Study ID	First author (Year)[Reference no.]	Origin of population	Sample size		miRNA profile	qPCR quantitation method
			Adenoma	Control		
I	Wu CW (2012)^20^	Hong Kong	57	88	miR-21	Absolute
II	Wu CW (2012)^20^	Hong Kong	57	88	miR-92a	Absolute
III	Wu CW (2012)^20^	Hong Kong	57	88	miR-21, miR-92a	Absolute
IV	Kanaoka S (2013)^22^	Japan	26	138	miR-21	Relative
V	Kanaoka S (2013)^22^	Japan	26	138	miR-92a	Relative
VI	Wu CW (2014)^25^	Hong Kong	169	104	miR-135b	Absolute
VII	Liu H (2016)^16^	China	120	98	miR-21	Relative
VIII	Liu H (2016)^16^	China	120	98	miR-146a	Relative
IX	Liu H (2016)^16^	China	120	98	miR-21, miR-146a	Relative
X	Wu CW (2017)^19^	USA	31	115	miR-451a, miR-144-5p	Relative

### Risk of bias

**Figure 2:**
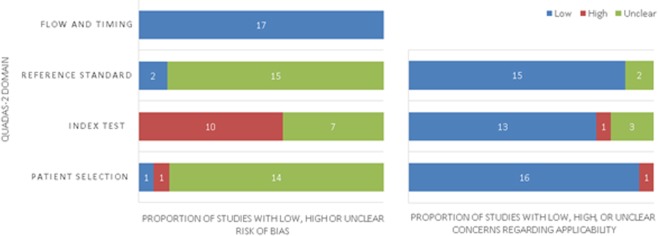
Quality assessment of included studies utilising the Quality Assessment of Diagnostic Accuracy Studies (QUADAS) version 2. Summary of risk of bias and applicability concerns for faecal-based miRNAs in the detection of colorectal cancer.

All included articles were evaluated for the risk of bias using the QUADAS-2 tool (Fig. 2)^30^. The major risk of bias in this study was in the index test, where 10 out of 17 publications had a high risk of bias due to the unclear or lack of statement regarding interpretation of index test results without knowledge of the results of the reference. Additionally, 14 out of 17 studies had an unclear risk of bias in the “Patient Selection” domain. This is due to a lack of detail on whether a consecutive or random sample of patients were enrolled. There was a low risk of bias in the reference standard, since all studies were histopathologically confirmed prior to the index test using either TNM or Dukes’ staging (Suppl. Table 1A,B). Concern about applicability in all domains was low.

### Pooled diagnostic accuracy of faecal-based microRNA for colorectal cancer and adenoma

**Figure 3:**
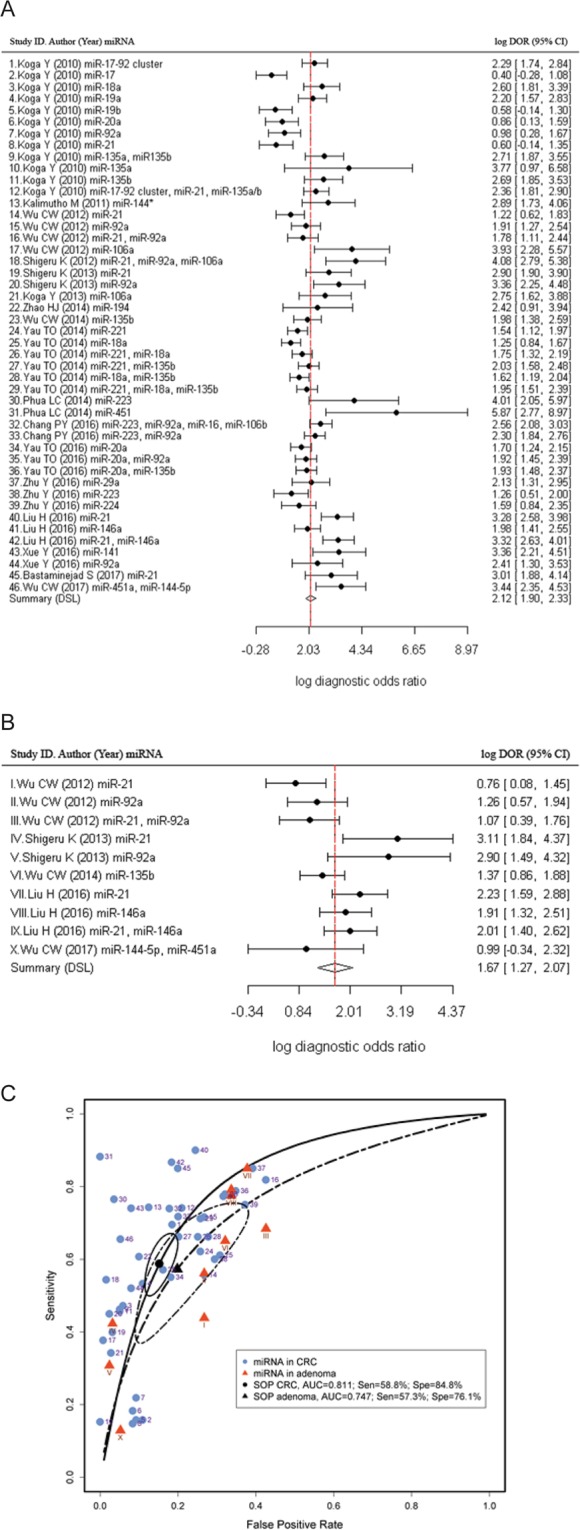
Diagnostic accuracy of pooled microRNAs in identification of colorectal cancer and colonic adenoma. Log Diagnostic Odds radios in (**A**) CRC was 2.12 (95% CI: 1.90–2.33) and (**B**) adenoma was 1.67 (95% CI: 1.27–2.07). (**C**) Summary receiver operating characteristic curves (SROC) for pooled miRNAs in CRC and colonic adenoma. The pooled miRNAs for CRC (n = 46) had a sensitivity of 58.8% (95% CI: 51.7–65.5%), specificity of 84.8% (95% CI: 81.1–87.8%) and AUC of 0.811. The pooled miRNAs for adenoma (n = 10) had a sensitivity of 57.3% (95% CI: 40.8–72.3%), specificity of 76.1% (95% CI: 66.1–89.4%) and AUC of 0.747. The number next to the dot/triangle corresponds to the study ID in Table 1 (Blue dots: CRC) or Table 2 (Red triangles: colonic adenoma). The circular regions (95% confidence contour) contain likely combinations of the mean value of sensitivity and specificity. Sen, sensitivity; Spe, specificity; SOP, summary operating point.

The detection accuracy of faecal-based miRNAs for CRC (Table 3) as well as colonic adenoma (Table 4) were pooled and analysed using the bivariate random effects model to evaluate the overall diagnostic measurements (Fig. 3). The DOR and log DOR were 8.32 (95% CI: 6.71–10.32) and 2.12 (95% CI: 1.90–2.33) for CRC and 5.31 (95% CI: 3.55–7.94) and 1.67 (95% CI: 1.27–2.07) for adenoma, respectively. The AUC value was 0.811 for the pooled CRC, and 0.747 for the pooled adenoma, respectively. The pooled studies of miRNAs in identification of CRC had a sensitivity of 58.8% (95% CI: 51.7–65.5%) and specificity of 84.8% (95% CI: 81.1–87.8%), whilst the pooled studies of miRNAs for identification of adenoma had a sensitivity of 57.3% (95% CI: 40.8–72.3%) and specificity of 76.1% (95% CI: 66.1–89.4%).

**Table 3:** Subgroup analysis for pooled microRNAs in the identification of CRC.

	No. of Studies	AUC	Partial AUC	log DOR (95% CI)	DOR (95% CI)	Sensitivity (95% CI)	Specificity (95% CI)	+LR (95% CI)	−LR (95% CI)	Meta-Regression	
										Sensitivity Z-value, *P*	Specificity Z-value, *P*
Pooled miRNAs for CRC	46	0.811	0.624	2.12 (1.90–2.33)	8.32 (6.71–10.32)	58.8% (51.7–65.5%)	84.8% (81.1–87.8%)	3.34 (2.93–3.81)	0.47 (0.42–0.53)	—	—
**Sample size**											
Small (Case n < 100)	14	0.801	0.681	2.32 (1.86–2.77)	10.16 (6.45–16.00)	70.6% (64.2–76.3%)	80.8% (72.3–87.1%)	3.38 (2.52–4.52)	0.38 (0.32–0.46)	2.458, *P* = 0.014	1.601, *P* = 0.109
Large (Case n > 100)	32	0.811	0.561	2.06 (1.81–2.31)	7.83 (6.11–10.04)	53.4% (44.4–62.3%)	86.3% (82.3–89.6%)	3.36 (2.90–3.90)	0.51 (0.45–0.57)		
**Pooled individual / Combination miRNAs**											
Individual miRNA	31	0.808	0.593	2.04 (1.73–2.35)	7.71 (5.65–10.52)	53.5% (43.8–62.9%)	86.4% (81.8–89.9%)	3.32 (2.76–4.00)	0.54 (0.48–0.60)	2.310, *P* = 0.021	1.284, *P* = 0.199
Combination miRNAs	15	0.801	0.674	2.28 (2.02–2.53)	9.73 (7.51–12.60)	68.8% (63.0–74.0%)	81.6% (75.0–86.8%)	3.47 (2.87–4.20)	0.39 (0.34–0.44)		
**Quantitation method**											
Absolute	13	0.763	0.685	1.74 (1.06–1.89)	5.706 (4.937–6.594)	67.3% (62.3–71.9%)	69.2% (69.4–77.0%)	2.49 (2.26–2.74)	0.46 (0.41–0.51)	−1.632, *P* = 0.103	−4.317, *P *< 0.001
Relative	33	0.846	0.662	2.37 (2.04–2.70)	10.738 (7.718–14.940)	55.5% (45.9–64.7%)	88.8% (85.2–91.6%)	4.27 (3.48–5.23)	0.49 (0.43–0.55)		

AUC, Area under curve; DOR, Diagnostic odds ratio; +LR, Positive likelihood ratio; −LR = Negative likelihood ratio; Z-value, regression coefficient. *P* < 0.05 was considered statistically significant.

**Table 4:** Subgroup analysis for pooled microRNAs in the identification of adenomas.

	No. of Studies	AUC	Partial AUC	log DOR (95% CI)	DOR (95% CI)	Sensitivity (95% CI)	Specificity (95% CI)	+LR (95% CI)	−LR (95% CI)	Meta-Regression	
										Sensitivity Z-value, *P*	Specificity Z-value, *P*
Pooled miRNAs for Adenoma	10	0.747	0.560	1.67 (1.27–2.07)	5.31 (3.55–7.94)	57.3% (40.8–72.3%)	76.1% (66.1–89.4%)	2.28 (1.84–2.84)	0.53 (0.41–0.68)	—	—
**Sample size**											
Small (Case n < 100)	4	0.647	0.490	1.53 (0.86–2.20)	6.36 (4.35–9.31)	42.0% (27.5–58.1%)	87.7% (70.0–95.6%)	2.23 (1.93–2.57)	0.35 (0.25–0.49)	−1.309 *P* < 0.001	−2.011 *P* = 0.044
Large (Case n > 100)	6	0.701	0.779	1.85 (1.47–2.23)	4.63 (2.37–9.04)	77.0% (67.7–84.2%)	65.7% (70.5–85.9%)	2.87 (1.71–4.83)	0.71 (0.59–0.85)		
**Pooled individual/Combination miRNAs**											
Individual miRNA	7	0.749	0.566	1.78 (1.26–2.30)	5.93 (3.52–10.00)	58.7% (41.6–73.9%)	81.6% (63.6–91.8%)	2.50 (1.86–3.36)	0.52 (0.40–0.67)	−0.185 *P* = 0.853	−0.194 *P* = 0.846
Combination miRNAs	3	0.729	0.520	1.45 (0.73–1.17)	4.26 (2.07–8.76)	52.6% (13.4–88.8%)	78.1% (42.2–94.5%)	1.97 (1.45–2.67)	0.55 (0.28–1.09)		
**Quantitation method**											
Absolute	4	0.687	0.626	1.16 (0.84–1.47)	3.18 (2.33–4.35)	59.4% (48.0–69.9%)	68.2% (36.7–52.0%)	1.83 (1.54–2.16)	0.61 (0.50–0.74)	−3.356 *P* = 0.001	−1.859 *P* = 0.063
Relative	6	0.802	0.622	2.12 (1.71–2.52)	8.32 (5.55–12.48)	56.9% (31.4–79.2%)	86.7% (67.6–95.3%)	3.01 (2.09–4.34)	0.48 (0.31–0.73)		

AUC, Area under curve; DOR, Diagnostic odds ratio; +LR, Positive likelihood ratio; −LR, Negative likelihood ratio; Z-value, regression coefficient. *P* < 0.05 was considered as statistically significant.

### Relative quantitation has a higher detection accuracy in colorectal cancer screening

To investigate the potential of faecal-based miRNA in the non-invasive diagnosis of CRC, studies were subgrouped in different aspects to compare their detection accuracy (Table 3). For the individual/combination miRNA analysis, both presented a similar power of diagnostic accuracy. The individual miRNA analysis panel had a sensitivity of 53.5% (95% CI: 43.8–62.9%), specificity of 86.4% (95% CI: 81.8–89.9%), DOR of 7.71 (5.65–10.52), and log DOR of 2.04 (95% CI: 1.73–2.35). The combination of miRNAs had a sensitivity of 68.8% (95% CI: 63.0–74.0%), specificity of 81.6% (95% CI: 75.0–86.8%), DOR of 9.73 (95% CI: 7.51–12.60), and log DOR of 2.28 (95% CI: 2.02–2.53). The AUC value was 0.808 for the individual miRNAs, and 0.801 for the combination of miRNAs, respectively. The meta-regression analysis showed a significant effect on pooled sensitivity (Z-value: 2.310, *P* = 0.021) but not in specificity (Z-value: 1.28, *P* = 0.199) (Table 4). Comparing studies with large (n > 100) versus small size (n ≤ 100), there was a sensitivity of 53.4% (95% CI: 44.4–62.3%) versus 70.6% (95% CI: 64.2–76.3%), specificity of 86.3% (95% CI: 82.3–89.6%) versus 80.8% (95% CI: 72.3–87.1%), DOR of 7.83 (95% CI: 6.11–10.04) versus 10.16 (95% CI: 6.45–16.00) and AUC of 0.811 versus 0.801 respectively. The meta-regression analysis indicated that the sample size did not significantly affect the pooled specificity (Z-value: 1.601, *P* = 0.109), however it did affect the pooled sensitivity (z-value: 2.458, *P* = 0.014).

The quantitation methods of absolute versus relative qPCR for faecal-based miRNAs were compared. The pooled relative quantitation qPCR method exhibited a better diagnostic accuracy in CRC (Table 3), with a sensitivity of 55.5% (95% CI: 45.9–64.7%), specificity of 88.8% (95% CI: 85.2–91.6%), DOR of 10.738 (95% CI: 7.718–14.940), log DOR of 2.37 (95% CI: 2.04–2.70) and AUC of 0.846. By contrast, the pooled absolute quantitation qPCR method exhibited sensitivity, specificity, DOR, log DOR and AUC of 67.3% (95% CI: 62.3–71.9%), 69.2% (95% CI: 69.4–77.0%), 5.706 (95% CI: 4.937–6.594), 1.74 (95% CI: 1.06–1.89) and 0.763, respectively. The meta-regression analysis revealed that the qPCR relative quantitation method in CRC affected only specificity (Z-value = −4.317, *P* < 0.001) when compared to the absolute quantification approach. The pooled relative quantitation qPCR approach exhibited a DOR of 8.32 (95% CI: 5.55–12.48) and log DOR of 2.12 (95% CI: 1.71–2.52) with a specificity of 86.7% (95% CI: 67.6–95.3%) and sensitivity of 56.9% (95% CI: 31.4–79.2%), compared with the absolute quantification approach (Table 3 and Suppl. Fig. 1).

Subgroup meta-regression analysis in colonic adenoma was also performed, looking at differences in sample size, pooled individual/combination miRNAs and quantitation method (Table 4). The meta-regression indicated that a small sample size significantly affected both the specificity (Z-value: −2.011, *P* = 0.044) and sensitivity (Z-value: −1.309, *P* < 0.001). With respect to the pooled individual/combination miRNAs, meta-regression analysis did not show significant effects in both the pooled sensitivity (Z-value: −10.85, *P* = 0.853) and specificity (Z-value: −0.194, *P* = 0.846). For the qPCR relative quantitation method, a significant effect was observed in the sensitivity (Z-value = −3.356, *P* < 0.001) of pooled miRNAs.

### Differences in detecting CRC depending on tumour stage and location

**Figure 4:**
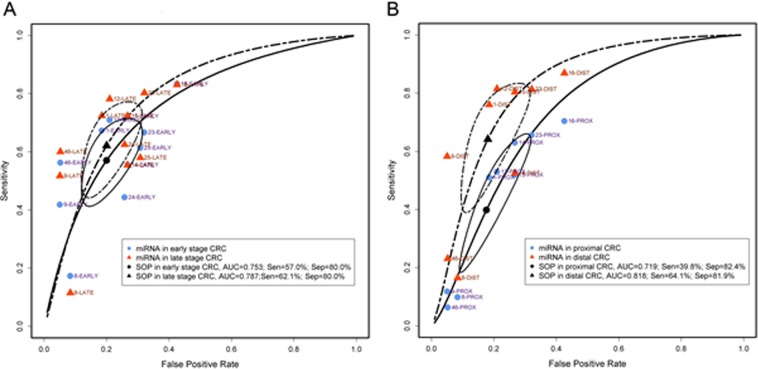
Diagnostic accuracy in early stage versus late stage and proximal versus distal colorectal cancer. (**A**) Summary receiver operating characteristic curves (SROC) for early (n = 11) and late (n = 11) stage CRC. (**B**) SROC for proximal (n = 9) and distal (n = 9) CRC. The number next to the blue dot/red triangle corresponds to the study ID in Table 1. The circular regions (95% confidence contour) contain likely combinations of the mean value of sensitivity and specificity. Sen = sensitivity; Spe = specificity SOP, summary operating point. ^#^Early stage CRC includes TMN stages 0 + I + II or Dukes’ stage A + B; late stage CRC includes CRC stages III + IV or Dukes’ stage C + D. Proximal CRC is defined as from cecum to transverse colon, and distal CRC is defined as from the splenic flexure to the rectum.

Meta-analysis on early versus late stage CRC as well as proximal versus distal CRC were performed to further evaluate the diagnostic ability of miRNAs. Pooled faecal miRNAs had a sensitivity of 57.0% (95% CI: 44.4–68.8%), specificity of 80.0% (95% CI: 71.1–86.7%), DOR of 5.58 (95% CI: 3.62–8.62) and log DOR of 1.72 (95% CI: 1.29–2.15) with respect to the diagnosis of early stage CRC, whilst in late stage CRC pooled miRNAs had a sensitivity of 62.1% (95% CI: 47.8–74.6%), specificity of 80.0% (95% CI: 71.1–86.7%), DOR of 6.70 (95% CI: 4.34–10.36) and log DOR of 1.90 (95% CI: 1.47–2.34) (Fig. 4A,B and Table 5). In proximal CRC, the pooled sensitivity, specificity, DOR, log DOR and AUC were 39.8% (95% CI: 21.8–61.0%), 82.4% (95% CI: 71.5–89.7%), 3.44 (95% CI: 2.53–4.66), 1.23 (95% CI: 0.93–1.54) and 0.719, respectively. For distal CRC, the pooled sensitivity, specificity, DOR, log DOR and AUC were 64.1% (95% CI: 43.9–80.3%), 81.9% (95% CI: 71.5–89.1%), 8.51 (95% CI: 4.97–14.57), 2.14 (95% CI: 1.60–2.68) and 0.818, respectively (Table 6 and Suppl. Fig. 2).

**Table 5:** Subgroup analysis for pooled microRNAs in association with CRC staging and tumour location.

miRNA	No. of Studies	AUC	Partial AUC	log DOR (95% CI)	DOR (95% CI)	Sensitivity (95% CI)	Specificity (95% CI)	+LR (95% CI)	−LR (95% CI)
**Staging#** (**Based on the data from the included studies**)									
Early	11	0.753	0.582	1.72 (1.29–2.15)	5.58 (3.62–8.62)	57.0% (44.4–68.8%)	80.0% (71.1–86.7%)	2.68 (2.10–3.43)	0.54 (0.43–0.68)
Late	11	0.787	0.627	1.90 (1.47–2.34)	6.70 (4.34–10.36)	62.1% (47.8–74.6%)	80.0% (71.1–86.7%)	2.82 (2.22–3.59)	0.45 (0.33–0.61)
**Tumour location** (**Based on the data from the included studies**)									
Proximal	9	0.719	0.488	1.23 (0.93–1.54)	3.44 (2.53–4.66)	39.8% (21.8–61.0%)	82.4% (71.5–89.7%)	2.08 (1.76–2.44)	0.75 (0.64–0.88)
Distal	9	0.818	0.675	2.14 (1.60–2.68)	8.51 (4.97–14.57)	64.1% (43.9–80.3%)	81.9% (71.5–89.1%)	3.04 (2.31–4.01)	0.41 (0.28–0.61)

DOR, Diagnostic odds ratio; +LR, Positive likelihood ratio; −LR, Negative likelihood ratio. ^#^Early stage CRC includes TNM stages 0 + I + II or Dukes’ stage A + B; Late stage CRC includes CRC stages III + IV or Dukes’ stage C + D.

**Table 6:** Diagnostic accuracy of individual microRNAs and microRNA combinations.

miRNA	Diagnosis	No. of Studies	AUC	Partial AUC	log DOR (95% CI)	DOR (95% CI)	Sensitivity (95% CI)	Specificity (95% CI)	+LR (95% CI)	−LR (95% CI)
miR-21	Adenoma	3	0.771	0.598	1.96 (1.96–3.23)	7.10 (1.99–25.34)	59.6% (27.7–85.0%)	83.0% (47.2–96.4%)	2.97 (1.45–6.07)	0.49 (0.27–0.90)
	CRC	5	0.843	0.549	2.23 (1.09–3.37)	9.28 (2.97–28.97)	59.3% (26.3–85.6%)	85.6% (72.2–93.2%)	3.38 (2.07–5.53)	0.43 (0.28–0.68)
miR-21-related combination	CRC	4	0.843	0.764	2.82 (1.95–3.69)	16.73 (7.00–39.94)	75.7% (60.3–86.5%)	85.0% (55.1–96.3%)	4.31 (2.22–8.39)	0.31 (0.20–0.46)
miR-92a	Adenoma	2	0.635	0.467	1.96 (0.36–3.55)	7.08 (1.43–34.97)	43.2% (20.1–69.8%)	91.0% (41.6–99.3%)	4.70 (0.80–27.60)	0.66 (0.54–0.81)
	CRC	4	0.794	0.537	2.15 (1.19–3.10)	8.57 (3.30–22.27)	46.8% (26.3–68.4%)	90.5% 77.1–96.4%)	4.53 (2.17–9.43)	0.57 (0.41–0.81)
miR-92a-related combination	CRC	5	0.791	0.685	2.35 (1.87–2.83)	10.47 (6.46–16.98)	68.2% (56.8–77.7%)	83.7% (65.9–93.2%)	3.71 (2.44–5.64)	0.40 (0.33–0.49)
miR-21 + miR-92a	CRC	9	0.837	0.548	2.19 (1.48–2.91)	8.97 (4.39–18.29)	53.7% (33.4–74.8%)	87.8% (79.5–93.0%)	3.68 (2.54–5.33)	0.51 (0.40–0.65)
miR-20a	CRC	2	0.797	0.367	1.35 (0.56–2.15)	3.87 (1.75–8.55)	34.4% (9.0–73.6%)	87.5% (73.6–94.6%)	2.84 (2.13–3.80)	0.70 (0.44–1.13)
miR-106a	CRC	2	0.416	0.356	3.38 (2.05–4.71)	29.33 (7.74–111.11)	36.1% (30.4–42.2%)	98.0% (94.7–99.2%)	18.85 (5.44–65.35)	0.65 (0.59–0.72)
miR-135b	CRC	2	0.798	0.656	2.32 (1.59–3.05)	10.18 (4.91–21.09)	63.1% 29.4–87.5%)	86.2% (41.8–98.2%)	4.44 (1.23–16.09)	0.44 (0.26–0.77)
miR-223	CRC	2	0.777	0684	2.69 (-0.42–5.79)	14.69 (0.66–328.44)	67.9% (47.9–83.0%)	87.4% (39.3–98.7%)	5.43 (0.56–52.59)	0.41 (0.18–0.92)

DOR, Diagnostic odds ratio; +LR, Positive likelihood ratio; −LR, Negative likelihood ratio.

### The detection accuracy of individual microRNAs

**Figure 5:**
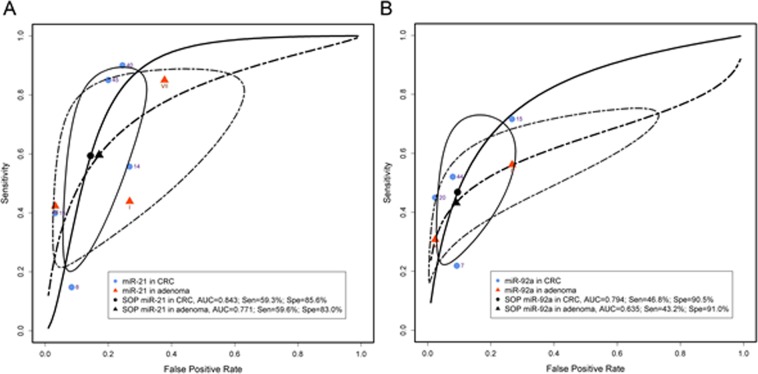
Diagnostic accuracy in pooled miR-21 and miR-92a. (**A**) SROC for pooled miR-21 in the detection of CRC (n = 5) and colonic adenoma (n = 3). (**B**) SROC for pooled miR-92a in the detection of CRC (n = 4) and colonic adenoma (n = 2). The number next to the dot/triangle corresponding to the study ID in Table 1 (Blue dots: CRC) or Table 2 (Red triangles: colonic adenoma). Sen, sensitivity; Spe, specificity; SOP, summary operating point. The circular regions (95% confidence contour) contain likely combinations of the mean value of sensitivity and specificity.

Each individual miRNA reported by more than one research group was pooled for an accuracy estimation (Table 6). miR-21 was reported by five CRC and three colonic adenoma studies^11,16,18,20,22^. Pooled miR-21 in CRC had an AUC of 0.843, DOR of 9.28 (95% CI: 2.97–28.97) and log DOR of 2.23 (95% CI: 1.09–3.37) whilst pooled miR-21 in adenoma had an AUC of 0.771, DOR of 7.10 (96% CI: 1.99–25.34) and log DOR of 1.96 (96% CI: 1.96–3.23) (Fig. 5A and Table 6). The miR-21-related combination pool for CRC detection had an AUC of 0.843, with a DOR of 16.73 (95% CI: 7.00–39.94) and log DOR of 2.82 (95% CI: 1.95–3.69) from four different CRC studies. miR-92a was reported in four CRC and two adenoma studies^11,20–22^. The AUC, DOR and log DOR were 0.794, 8.57 (95% CI: 3.30–22.27) and 2.15 (95% CI: 1.19–3.10) for pooled miR-92a alone in the diagnosis of CRC, and 0.635, 0.467, 7.08 (95% CI: 1.43–34.97) and 1.96 (95% CI: 0.36–3.55) for pooled miR-92a alone in the diagnosis of colonic adenoma, respectively (Fig. 5B and Table 6).

The miR-92a-related combination pool for CRC was reported in five CRC studies, with AUC 0.791, DOR 10.47 (95% CI: 6.46–16.98) and log DOR 2.35 (95% CI: 1.87–2.83). The pooled miR-21 plus miR-92a combination exhibited an AUC of 0.837, with a specificity of 87.8% (95% CI: 79.5–93.0%), sensitivity of 53.7% (95% CI: 33.4–74.8%) and DOR of 2.19 (95% CI: 1.48–2.91). miR-20a, miR-106a, miR-135b and miR-223 were reported in two different articles, with an AUC of 0.797, 0.416, 0.798 and 0.777, DOR of 3.87 (95% CI: 1.75–8.55), 29.33 (95% CI: 7.74–111.11), 10.18 (95% CI: 4.91–21.09) and 14.69 (95% CI: 0.66–328.44), respectively (Table 6 and Suppl. Fig. 2).

### Publication bias evaluation

**Figure 6:**
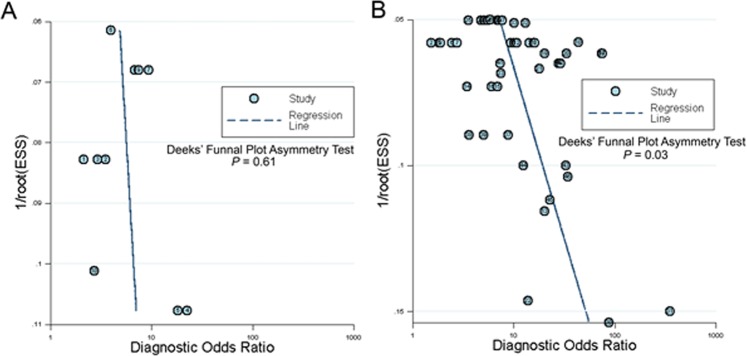
Deeks’ funnel plot asymmetry test for the assessment of potential bias in microRNA assays. (**A**) Pooled miRNAs for CRC and, (**B**) Pooled miRNAs for colonic adenoma.

The Deeks’ funnel plot asymmetry test was utilised to evaluate the potential publication bias from each included faecal-based miRNA study. The slope coefficient was associated with *P* = 0.03 in the pooled miRNAs in CRC, and *P* = 0.61 in pooled miRNAs in colonic adenoma studies, indicating that significant asymmetry was found in the CRC dataset (Fig. 6A) but not in the colonic adenoma dataset (Fig. 6B). The combination of CRC and colonic adenoma resulted in a *P* = 0.11 (Suppl. Fig. 3).

## Discussion

To evaluate the diagnostic value of faecal-based miRNAs, data from 17 eligible publications, including 46 studies on miRNAs in CRC and 10 studies on colonic adenoma, corresponding to 6475, 783 and 5569 faecal-based miRNA tests in CRC patients, adenoma and healthy control volunteers, respectively, were subjected to meta-analysis. Our study reveals that pooled faecal-based miRNAs have a relatively high detection accuracy for CRC. However, the lack of consensus regarding the optimal quantitation method, data normalisation, and selection of control subjects, may present obstacles to clinical application.

qPCR-based studies were the subject of our meta-analysis analysis. This approach for miRNA level quantification is advantageous compared to others in that is fast and easily adoptable in a clinical setting. However, it comes with limitations that relate to the method for miRNA isolation and the selection of the appropriate reference/normalisation control. Although reference quantitation method demonstrated a better diagnostic accuracy compared to absolute quantitation, it is important to acknowledge that a variety of internal controls were used as references, including RUN6B(U6)^11,15–18,24^, miR-24^23^, miR-200b-3p^19^, miR-378^12^, miR-1202^27^ and miR-4257^27^. Increasing evidence suggests that RUN6B may not be a suitable endogenous control for miRNAs^31,32^ due to its rapid degradation in faeces^20^. miRNAs used as internal controls also have functions in the host cell, and their deregulation could interfere with the detection accuracy. For example, miR-24, a proposed tumour suppressor miRNA in CRC, controls cellular proliferation independently of p53 by targeting the 3’UTR of dihydrofolate reductase (DHFR) mRNA^33,34^. Deregulation of plasma miR-378 was also found in CRC patients^35^. miR-4257 has been reported to be down-regulated in bladder cancer cell lines and up-regulated in the plasma of patients with recurrence of non-small cell lung cancer (NSCLC)^36,37^. Peripheral levels of miR-1202 predicts and mediates the response to anti-depressants, specifically regulating the expression of metabotropic glutamate receptor-4 (GRM4) with levels correlating to changes in brain activity^38–40^. miR-1202 is deregulated in different types of cancers, such as breast cancer^41^, gastric cancer^42^ and clear cell papillary renal cell carcinoma^43^. Absolute quantitation was employed in several studies for faecal-based miRNA screening^13,14,20,26^ (Table 1), however, this necessitates a standard curve which depends on the quantification detection method and does not eliminate potential contamination by gut bacteria DNA/RNA^14^.

The combination of FIT and stool-based miRNA markers may increase detection accuracy to overcome this problem. A previous study indicated that the combination of miR-21 and miR-92a with FIT had a specificity of 96.8% and sensitivity of 78.4% while FIT alone only had a specificity of 98.4% and sensitivity of 66.7%^22^. A parameter that should be considered is the presence/absence of occult blood in samples as miRNAs expressed in blood cells may interfere with the assay, altering the levels of specific miRNAs. In an effort to assess the potential contribution of blood in faecal miRNA levels we have retrieved a list of circulating miRNAs^44^. Comparison showed that 8 miRNAs are detected in both blood and faecal specimens (Suppl. Fig. 4). This finding does not imply that blood cells are responsible for the alterations in the levels of these miRNAs, as their origin may as well be the tumour. Optimally, a controlled study including comparisons between samples positive and negative for FOBT/FIT could address the relative contribution. A more inclusive approach employing miRNA analyses and comparisons between matched blood, faecal specimens and tumours or colonic tissues would be most informative about the source of changes in miRNA levels. Furthermore, other colonic pathologies like inflammatory bowel diseases are characterised by deregulation of miRNAs detectable in tissues and serum^45–48^ and the presence of occult blood in faeces. A comprehensive analysis would include samples from different pathologies of the colon, assess and identify disease-specific miRNA signatures and their diagnostic/prognostic properties.

A clear conclusion on which quantitation method is more suitable cannot be drawn with the currently available data. The use of multiple internal controls or the geometric mean, using a multiplex screening method, such as microarrays or next generation sequencing, would provide the optimal means of normalisation. Alternatively, the NanoString nCounter technology enables profiling of around 800 molecular targets in one single reaction by utilising molecular “barcodes”. This approach normalises the data by using multiple targets, and more importantly quantifies multiple miRNAs which can be used simultaneously as biomarkers to improve detection accuracy. In addition, this platform overcomes the need for data processing and bioinformatic analysis expertise, as in the case of microarrays or high-throughput sequencing, thus may be easily utilised in a clinical setting^46^.

To evaluate the potential detection efficiency for each individual miRNA, individual miRNAs reported in more than one study were grouped to evaluate its detection accuracy. In this meta-analysis, miR-21 and miR-92a were the most commonly reported faecal-based miRNAs (Table 6). Numerous studies have characterised the functional roles of these two miRNAs in CRC pathogenesis and aggressiveness. Up-regulation of miR-21 and miR-92a promotes CRC cell migration, invasion and proliferation^11,16,18,20,22^, and inhibition of apoptosis^49–51^. Several significant targets of miR-21 are associated with CRC malignancy, such as phosphatase and tensin homolog (PTEN)^49,52^, programmed cell death protein 4 (PDCD4)^53,54^, and *ras* homolog gene family member B (RhoB)^55^. Among these, PTEN was reported frequently silenced in CRC by miR-21, resulting in PI3K/AKT pathway activation and induction of tumour formation^49,52^. Recently, a long non-coding RNA (LINC00312) suppressed in CRC was shown to regulate miR-21 levels through its function as a miRNA sponge, thereby regulating PTEN expression^56^. miR-92a has been shown to disrupt the expression of several tumour suppressors such as PTEN^57,58^, Dickkopf WNT Signalling Pathway Inhibitor 3 (DKK3)^57^, Kruppel-like factor 4 (KLF4)^59^ and mothers against decapentaplegic homolog 7 (SMAD7)^60^. Hence, miR-92a activates the PI3K/AKT, WNT/β-catenin and BMP/Smad pathways and enhances tumorigenesis. Subject to this analysis five studies reported the use of miR-21 in the identification of CRC, and three studies reported its use in identification of adenomas^11,16,18,20,22^. Four studies reported the utility of miR-92a in the identification of CRC, and two studies in identification of adenomas^11,17,20,22^. miR-21 had a better detection accuracy range compared with miR-92a, with a DOR of 9.28 (95% CI: 2.97–28.97) and summary AUC of 0.843. Panels including a combination of either miR-21 or miR-92a, as well as panels including both miR-21 and miR-92a demonstrated a small improvement in detection (Fig. 5 and Table 6). However, due to the small number of published studies, with each having wide confidence intervals, a direct comparison between two faecal-based miRNAs may not be accurate. Additional data are needed to limit potential errors.

The FOBT or FIT, have limited sensitivity for detecting proximal compared with distal CRC^61,62^. This is due to the degradation of haemoglobin. Hence, tumour location analysis for faecal-based miRNA detection was also considered and reported by several studies – with none of them reporting a statistical difference. In this study, the results between pooled miRNAs for proximal and distal CRC reveal differences associated with tumour location, with an AUC of 0.719 versus 0.818, and DOR of 3.44 (95% CI: 2.53–4.66) versus 8.51 (95% CI: 4.97–14.57) (Fig. 4B and Table 5).

Our study is characterised by many strengths but should be interpreted in the context of specific shortcomings. Firstly, subgroup analysis suggested that the combination of faecal miRNAs exhibited a good accuracy for CRC and colonic adenoma patients screening (Tables 3, 4 and Fig. 3). However, certain combinations of miRNAs may not significantly improve the detection accuracy. For example, the panel containing miR-223, miR-92a, miR-16 and miR-106b had a sensitivity of 73.9%, specificity of 82.2% and AUC of 0.84^13^, whereas the combination of miR-18a and miR-135b only had a sensitivity of 66%, specificity of 72% and AUC of 0.75^26^. Therefore, an optimal miRNA combination panel should be prioritised. Secondly, the majority of studies were performed in East Asia (Hong Kong, Taiwan, China, Japan and Singapore) (Table 1) with only one study in the USA, Europe and the Middle East, making it unclear whether the ethnic background of participants has an influence on the expression of miRNAs in CRC. Thirdly, due to the high cost of colonoscopy, the majority of test subjects were recruited from the corresponding clinics. This may result in a degree of bias, since the subjects are not representative of the general population. Last but not least, the publication bias analysis revealed that pooled miRNAs in CRC have a significant asymmetry (*P* = 0.03). This may be due to file-drawer effects, bias from the studies with small same sizes, lack of clarity in reporting the results for some publications, or the level of detail provided being lower than the one required for our analysis. Consequently, some studies were excluded, resulting in a possible bias in our meta-analysis (Fig. 6A).

In conclusion, faecal-based miRNAs show a relatively high accuracy for the non-invasive detection of colonic adenomas and CRC in the studied population. The use of a panel of miRNAs as biomarkers may result in a higher CRC detection rate, while the combination of miRNA biomarkers with FOBT or FIT may increase the detection accuracy. Large, ideally multi-centre, double-blinded randomised controlled trials are needed to establish the value of miRNAs as biomarkers in CRC screening within the general population.

## Methods

### Overview

The study protocol followed the Cochrane Handbook for Diagnostic Test Accuracy Review^63^ and the Preferred Reporting Items in Systematic Reviews and the Meta-Analysis statement (PRISMA)^64^. Investigators of each of the original studies obtained approval from their local ethics committee and had written, informed patient consent.

### Literature search strategy

The search strategy was designed to identify any studies describing the diagnostic value of faecal-based miRNA for CRC and colonic adenoma patients. After an initial search for articles in PubMed, assessments of key terms within the title and abstract were conducted. A full systematic search using the established key terms was adopted for the following databases: PubMed, Ovid Embase, The Cochrane Library, Scopus and Web of Science. The search terms used were “miRNA OR microRNA OR miR” AND “colorectal cancer OR colorectal tumor OR colorectal adenocarcinoma OR colorectal carcinoma OR colorectal neoplasm OR colon cancer OR colonic adenoma OR colonic adenocarcinoma OR stomach cancer OR rectal cancer OR CRC” AND “stool OR feces”. The search formulas are available as supplementary data (Supplementary data 1). Manual searching of related citations and reference lists was undertaken. Book chapters, letters to editors, commentaries, editorials, patents, and non-peer reviewed articles were excluded. Two investigators independently screened the search results, initially through articles’ title and abstract. The filtered candidate articles were then scrutinised independently through full-text reading. Discrepancies were resolved through discussion between the two investigators.

### Study selection criteria

All research articles in any language published up to November 17, 2017 were eligible for inclusion. An electronic data extraction form was developed, and pre-tested, with data extracted by two researchers. Eligible studies included in this meta-analysis adhered to the following criteria: (1) studies evaluated the diagnostic value of miRNAs for detecting human CRC or colonic adenomas; (2) all CRC patients involved in the study had been confirmed by histology; (3) studies contained data on miRNAs’ sensitivity, specificity, and sample size to enable reconstruction of the diagnostic 2 × 2 contingency table. Exclusion criteria were set as follows: (1) duplicated studies, the later ones were excluded; (2) publications that were unrelated to the diagnostic value of miRNAs for CRC; (3) incomplete data reporting. The detection accuracy of miRNAs between proximal (from cecum to transverse colon) and distal (from splenic flexure to rectum) CRC, as well as between early (CRC stages 0 + I + II or Dukes’ stages A + B) and late (CRC stages III + IV or Dukes’ stages C + D) stage CRC were evaluated separately if investigators reported the location and stage. Each individual miRNA and miRNA combinations were grouped together if found in more than two studies.

### Risk of bias

The Quality Assessment of Diagnostic Accuracy Studies-2 (QUADAS-2) was utilised to assess the quality of included publications, evaluating four key domains (“Patient Selection”, “Index Test”, “Reference Standard”, and “Flow and Timing”) in two categories (risk of bias and applicability of diagnostic accuracy studies)^30^. Each category in all publications was judged as low, high or unclear based on the assessment criteria provided. Assessment of each included study was performed by two investigators, with disagreements resolved by consensus after discussion.

### Data synthesis

A meta-analysis of diagnostic test accuracy was conducted on faecal-based non-invasive miRNA tests through a bivariate random effects modelling approach. The bivariate model accounts for the correlation between the studies’ sensitivity and specificity in two different levels. The first level represents a variability between sensitivity and specificity within one study; the second level represents the heterogeneity in diagnostic performance of the index test across the testing studies. Random effects meta-analysis methods were applied in our study as heterogeneity is presumed to exist.

Statistical analyses in this study were performed using the statistical package mada version 0.4.8 in R (version 3.4.3) to implement the bivariate normal approach of Reitsma *et al*.^65^. Sensitivity, specificity, diagnostic odds ratio (DOR), log DOR, positive likelihood ratio (+LR) and negative likelihood ratio (–LR) were calculated along with their 95% confidence interval (95% CI) based on the random effects model (DerSimonian and Laird method) with continuity correction^66,67^. Summary receiver operating characteristic (sROC) curves, area under the curve (AUC) and partial AUC were also utilised to examine the pooled faecal-based miRNAs in CRC, adenoma and the subgroups. Potential sources of heterogeneity were investigated using subgroup and bivariate meta-regression (restricted maximum likelihood (REML) estimators) analysis. The Deeks’ funnel plot asymmetry test was examined using the midas package in Stata (version 12).

### Interpretation of diagnostic test accuracy statistics

The AUC was interpreted in four-grades: >0.97, excellent; 0.93–0.96, very good; 0.75–0.92, good; < 0.75, not accurate^68^. The values of –LR and +LR were also divided into four categories. The –LR values < 0.1, 0.1–0.2, 0.2–0.5 and >0.5 were identified as large, moderate, small and not meaningful decreases in probability, respectively^69^. The +LR values >10, 5–10, 2–5 and <2 were classified as large, moderate, small and not meaningful increases in probability, respectively^69^. *P* < 0.05 was considered statistically significant.

## Supplementary information

Supplementary Data
