# Supplementary material for: Faecal microRNAs as a non-invasive tool in the diagnosis of colonic adenomas and colorectal cancer: A meta-analysis
Source: Sci Rep. 2019 Jul 1;9:9491. doi: 10.1038/s41598-019-45570-9 (PMC6603164; doi:10.1038/s41598-019-45570-9)
Supplement: Supplementary file 1 — Supplementary Data [file 41598_2019_45570_MOESM1_ESM.docx]

**Faecal microRNAs as a non-invasive tool in the diagnosis of colonic adenomas and colorectal cancer: A meta-analysis**

Tung On Yau^1^, Ceen-Ming Tang^2^, Elinor K Harriss^3^, Benjamin Dickins^1^, Christos Polytarchou^1^*****

**1.** Biosciences, John van Geest Cancer Research Centre, School of Science and Technology, Nottingham Trent University, Nottingham, UK

**2.** Oxford University Clinical Academic Graduate School, John Radcliffe Hospital, Oxford, UK

**3.** Bodleian Health Care Libraries, University of Oxford, Oxford, UK

**Supplementary Information**

**Supplementary Table 1A.** Sample collection procedure and clinical data reported for all included colorectal cancer studies.

| CRC | Author | Year | miRNA | Histology | History of IBD | FIT/ FOBT | CRC - Collection | Control - Collection |
| --- | --- | --- | --- | --- | --- | --- | --- | --- |
| 1 | Koga Y | 2010 | miR-17-92 cluster | Y | unknown | unknown | before surgical resection | few weeks after colonoscopy |
| 2 | Koga Y | 2010 | miR-17 | Y | unknown | unknown | before surgical resection | few weeks after colonoscopy |
| 3 | Koga Y | 2010 | miR-18a | Y | unknown | unknown | before surgical resection | few weeks after colonoscopy |
| 4 | Koga Y | 2010 | miR-19a | Y | unknown | unknown | before surgical resection | few weeks after colonoscopy |
| 5 | Koga Y | 2010 | miR-19b | Y | unknown | unknown | before surgical resection | few weeks after colonoscopy |
| 6 | Koga Y | 2010 | miR-20a | Y | unknown | unknown | before surgical resection | few weeks after colonoscopy |
| 7 | Koga Y | 2010 | miR-92a | Y | unknown | unknown | before surgical resection | few weeks after colonoscopy |
| 8 | Koga Y | 2010 | miR-21 | Y | unknown | unknown | before surgical resection | few weeks after colonoscopy |
| 9 | Koga Y | 2010 | miR-135a, miR135b | Y | unknown | unknown | before surgical resection | few weeks after colonoscopy |
| 10 | Koga Y | 2010 | miR-135a | Y | unknown | unknown | before surgical resection | few weeks after colonoscopy |
| 11 | Koga Y | 2010 | miR-135b | Y | unknown | unknown | before surgical resection | few weeks after colonoscopy |
| 12 | Koga Y | 2010 | miR-17-92 cluster, miR-21, miR-135a/b | Y | unknown | unknown | before surgical resection | few weeks after colonoscopy |
| 13 | Kalimutho M | 2011 | miR-144* | Y | unknown | unknown | before surgical resection | before colonoscopy |
| 14 | Wu CW | 2012 | miR-21 | Y | excluded | unknown | 2 weeks before surgical resection | asymptomatic - from screening programme |
| 15 | Wu CW | 2012 | miR-92a | Y | excluded | unknown | 2 weeks before surgical resection | asymptomatic - from screening programme |
| 16 | Wu CW | 2012 | miR-21, miR-92a | Y | excluded | unknown | 2 weeks before surgical resection | asymptomatic - from screening programme |
| 17 | Shigeru K | 2012 | miR-106a | Y | unknown | FIT | before surgical resection | not stated - collected from hospital |
| 18 | Shigeru K | 2012 | miR-21, miR-92a, miR-106a | Y | unknown | FIT | before surgical resection | not stated - collected from hospital |
| 19 | Shigeru K | 2013 | miR-21 | Y | unknown | FIT | before surgical resection | not stated - collected from hospital |
| 20 | Shigeru K | 2013 | miR-92a | Y | unknown | FIT | before surgical resection | not stated - collected from hospital |
| 21 | Koga Y | 2013 | miR-106a | Y | unknown | FIT | before surgical resection | few weeks after colonoscopy |
| 22 | Zhao HJ | 2014 | miR-194 | Y | unknown | unknown | not stated - collected from hospital | not stated - collected from hospital |
| 23 | Wu CW | 2014 | miR-135b | Y | excluded | unknown | 2 weeks before surgical resection | asymptomatic - from screening programme |
| 24 | Yau TO | 2014 | miR-221 | Y | excluded | unknown | 2 weeks before surgical resection | asymptomatic - from screening programme |
| 25 | Yau TO | 2014 | miR-18a | Y | excluded | unknown | 2 weeks before surgical resection | asymptomatic - from screening programme |
| 26 | Yau TO | 2014 | miR-221, miR-18a | Y | excluded | unknown | 2 weeks before surgical resection | asymptomatic - from screening programme |
| 27 | Yau TO | 2014 | miR-221, miR-135b | Y | excluded | unknown | 2 weeks before surgical resection | asymptomatic - from screening programme |
| 28 | Yau TO | 2014 | miR-18a, miR-135b | Y | excluded | unknown | 2 weeks before surgical resection | asymptomatic - from screening programme |
| 29 | Yau TO | 2014 | miR-221, miR-18a, miR-135b | Y | excluded | unknown | 2 weeks before surgical resection | asymptomatic - from screening programme |
| 30 | Phua LC | 2014 | miR-223 | Y | excluded | unknown | before surgical resection | few weeks after colonoscopy |
| 31 | Phua LC | 2014 | miR-451 | Y | excluded | unknown | before surgical resection | few weeks after colonoscopy |
| 32 | Chang PY | 2016 | miR-223, miR-92a, miR-16, miR-106b | Y | excluded | unknown | collected prior to surgery | self-reported - general population |
| 33 | Chang PY | 2016 | miR-223, miR-92a | Y | excluded | unknown | collected prior to surgery | self-reported - general population |
| 34 | Yau TO | 2016 | miR-20a | Y | excluded | unknown | 2 weeks before surgical resection | asymptomatic - from screening programme |
| 35 | Yau TO | 2016 | miR-20a, miR-92a | Y | excluded | unknown | 2 weeks before surgical resection | asymptomatic - from screening programme |
| 36 | Yau TO | 2016 | miR-20a, miR-135b | Y | excluded | unknown | 2 weeks before surgical resection | asymptomatic - from screening programme |
| 37 | Zhu Y | 2016 | miR-29a | Y | excluded | unknown | not stated - collected from hospital | not stated - collected from hospital |
| 38 | Zhu Y | 2016 | miR-223 | Y | excluded | unknown | not stated - collected from hospital | not stated - collected from hospital |
| 39 | Zhu Y | 2016 | miR-224 | Y | excluded | unknown | not stated - collected from hospital | not stated - collected from hospital |
| 40 | Liu H | 2016 | miR-21 | Y | unknown | unknown | before surgical resection | not stated - collected from hospital |
| 41 | Liu H | 2016 | miR-146a | Y | unknown | unknown | before surgical resection | not stated - collected from hospital |
| 42 | Liu H | 2016 | miR-21, miR-146a | Y | unknown | unknown | before surgical resection | not stated - collected from hospital |
| 43 | Xue Y | 2016 | miR-141 | Y | unknown | unknown | before surgical resection | asymptomatic - from screening |
| 44 | Xue Y | 2016 | miR-92a | Y | unknown | unknown | before surgical resection | asymptomatic - from screening |
| 45 | Bastaminejad S | 2017 | miR-21 | Y | excluded | YES - all negative | before surgical resection | clinical history + FOBT/FIT negative |
| 46 | Wu CW | 2017 | miR-451a, miR-144-5p | Y | unknown | YES | before surgical resection | not stated - collected from hospital |

**Supplementary Table 1B.** Sample collection procedure and clinical data reported for all included colonic adenoma studies.

| Adenoma | Author | Year | miRNA | Histology | History of IBD | FIT/ FOBT | Colonic Adenoma - Collection | Control - Collection |
| --- | --- | --- | --- | --- | --- | --- | --- | --- |
| I | Wu CW | 2012 | miR-21 | Y | excluded | unknown | 2 weeks before surgical resection | asymptomatic - from screening programme |
| II | Wu CW | 2012 | miR-92a | Y | excluded | unknown | 2 weeks before surgical resection | asymptomatic - from screening programme |
| III | Wu CW | 2012 | miR-21, miR-92a | Y | excluded | unknown | 2 weeks before surgical resection | asymptomatic - from screening programme |
| IV | Shigeru K | 2013 | miR-21 | Y | unknown | FIT | before surgical resection | not stated - collected from hospital |
| V | Shigeru K | 2013 | miR-92a | Y | unknown | FIT | before surgical resection | not stated - collected from hospital |
| VI | Wu CW | 2014 | miR-135b | Y | excluded | unknown | 2 weeks before surgical resection | asymptomatic - from screening programme |
| VII | Liu H | 2016 | miR-21 | Y | unknown | unknown | before surgical resection | not stated - collected from hospital |
| VIII | Liu H | 2016 | miR-146a | Y | unknown | unknown | before surgical resection | not stated - collected from hospital |
| IX | Liu H | 2016 | miR-21, miR-146a | Y | unknown | unknown | before surgical resection | not stated - collected from hospital |
| X | Wu CW | 2017 | miR-451a, miR-144-5p | Y | unknown | YES | before surgical resection | not stated - collected from hospital |

**Supplementary Table 2A.** miRNA extraction and qPCR quantitation methods reported in all included colorectal cancer studies.

| CRC | Author | Year | miRNA | miRNA Panel | Internal control | qPCR quantitation method | Multi- miRNA test | miRNA extraction method |
| --- | --- | --- | --- | --- | --- | --- | --- | --- |
| 1 | Koga Y | 2010 | miR-17-92 cluster | Y | RUN6B | Relative | Y | RNeasy Mini Kit (Qiagen) |
| 2 | Koga Y | 2010 | miR-17 | N | RUN6B | Relative | Y | RNeasy Mini Kit (Qiagen) |
| 3 | Koga Y | 2010 | miR-18a | N | RUN6B | Relative | Y | RNeasy Mini Kit (Qiagen) |
| 4 | Koga Y | 2010 | miR-19a | N | RUN6B | Relative | Y | RNeasy Mini Kit (Qiagen) |
| 5 | Koga Y | 2010 | miR-19b | N | RUN6B | Relative | Y | RNeasy Mini Kit (Qiagen) |
| 6 | Koga Y | 2010 | miR-20a | N | RUN6B | Relative | Y | RNeasy Mini Kit (Qiagen) |
| 7 | Koga Y | 2010 | miR-92a | N | RUN6B | Relative | Y | RNeasy Mini Kit (Qiagen) |
| 8 | Koga Y | 2010 | miR-21 | N | RUN6B | Relative | Y | RNeasy Mini Kit (Qiagen) |
| 9 | Koga Y | 2010 | miR-135a, miR135b | Y | RUN6B | Relative | Y | RNeasy Mini Kit (Qiagen) |
| 10 | Koga Y | 2010 | miR-135a | N | RUN6B | Relative | Y | RNeasy Mini Kit (Qiagen) |
| 11 | Koga Y | 2010 | miR-135b | N | RUN6B | Relative | Y | RNeasy Mini Kit (Qiagen) |
| 12 | Koga Y | 2010 | miR-17-92 cluster, miR-21, miR-135a/b | Y | RUN6B | Relative | Y | RNeasy Mini Kit (Qiagen) |
| 13 | Kalimutho M | 2011 | miR-144* | N | mean expression of miR-378 in total samples | Relative | Y | mirVana™ miRNA isolation kit |
| 14 | Wu CW | 2012 | miR-21 | N | - | Absolute | Y | RNeasy Mini Kit (Qiagen) |
| 15 | Wu CW | 2012 | miR-92a | N | - | Absolute | Y | RNeasy Mini Kit (Qiagen) |
| 16 | Wu CW | 2012 | miR-21, miR-92a | Y | - | Absolute | Y | RNeasy Mini Kit (Qiagen) |
| 17 | Shigeru K | 2012 | miR-106a | N | custom miRNA | Relative | Y | unknown |
| 18 | Shigeru K | 2012 | miR-21, miR-92a, miR-106a | Y | custom miRNA | Relative | Y | unknown |
| 19 | Shigeru K | 2013 | miR-21 | N | custom miRNA | Relative | Y | unknown |
| 20 | Shigeru K | 2013 | miR-92a | N | custom miRNA | Relative | Y | unknown |
| 21 | Koga Y | 2013 | miR-106a | N | miR-24 | Relative | Y | miRNeasy Mini Kit (Qiagen) |
| 22 | Zhao HJ | 2014 | miR-194 | N | RUN6B | Relative | N | Trizol |
| 23 | Wu CW | 2014 | miR-135b | N | - | Absolute | Y | RNeasy Mini Kit (Qiagen) |
| 24 | Yau TO | 2014 | miR-221 | N | - | Absolute | Y | RNeasy Mini Kit (Qiagen) |
| 25 | Yau TO | 2014 | miR-18a | N | - | Absolute | Y | RNeasy Mini Kit (Qiagen) |
| 26 | Yau TO | 2014 | miR-221, miR-18a | Y | - | Absolute | Y | RNeasy Mini Kit (Qiagen) |
| 27 | Yau TO | 2014 | miR-221, miR-135b | Y | - | Absolute | Y | RNeasy Mini Kit (Qiagen) |
| 28 | Yau TO | 2014 | miR-18a, miR-135b | Y | - | Absolute | Y | RNeasy Mini Kit (Qiagen) |
| 29 | Yau TO | 2014 | miR-221, miR-18a, miR-135b | Y | - | Absolute | Y | RNeasy Mini Kit (Qiagen) |
| 30 | Phua LC | 2014 | miR-223 | N | miR-1202, miR-4257 | Relative | Y | mirVana™ miRNA isolation kit |
| 31 | Phua LC | 2014 | miR-451 | N | miR-1202, miR-4257 | Relative | Y | mirVana™ miRNA isolation kit |
| 32 | Chang PY | 2016 | miR-223, miR-92a, miR-16, miR-106b | Y | Spike-in cel-miR-238 | Relative | Y | RNeasy Mini Kit (Qiagen) |
| 33 | Chang PY | 2016 | miR-223, miR-92a | Y | Spike-in cel-miR-238 | Relative | Y | RNeasy Mini Kit (Qiagen) |
| 34 | Yau TO | 2016 | miR-20a | N | Absolute | Absolute | Y | RNeasy Mini Kit (Qiagen) |
| 35 | Yau TO | 2016 | miR-20a, miR-92a | Y | - | Absolute | Y | RNeasy Mini Kit (Qiagen) |
| 36 | Yau TO | 2016 | miR-20a, miR-135b | Y | - | Absolute | Y | RNeasy Mini Kit (Qiagen) |
| 37 | Zhu Y | 2016 | miR-29a | N | RUN6B + Reference control samples | Relative | Y | stool RNA extraction kit (Omega |
| 38 | Zhu Y | 2016 | miR-223 | N | RUN6B + Reference control samples | Relative | Y | stool RNA extraction kit (Omega |
| 39 | Zhu Y | 2016 | miR-224 | N | RUN6B + Reference control samples | Relative | Y | stool RNA extraction kit (Omega |
| 40 | Liu H | 2016 | miR-21 | N | RUN6B | Relative | Y | TRIzol |
| 41 | Liu H | 2016 | miR-146a | N | RUN6B | Relative | Y | TRIzol |
| 42 | Liu H | 2016 | miR-21, miR-146a | Y | RUN6B | Relative | Y | TRIzol |
| 43 | Xue Y | 2016 | miR-141 | N | RUN6B | Relative | Y | TRIzol |
| 44 | Xue Y | 2016 | miR-92a | N | RUN6B | Relative | Y | TRIzol |
| 45 | Bastaminejad S | 2017 | miR-21 | N | Absolute,spike-in control, RUN6B | Relative | N | RNeasy Mini Kit (Qiagen) |
| 46 | Wu CW | 2017 | miR-451a, miR-144-5p | Y | miR-200b-3p | Relative | Y | RNeasy Mini Kit (Qiagen) |

**Supplementary Table 2B.** miRNA extraction and qPCR quantitation methods reported in all included adenoma studies.

| Adenoma | Author | Year | miRNA | miRNA Panel | Internal control | qPCR quantitation method | Multi- miRNA test | miRNA extraction method |
| --- | --- | --- | --- | --- | --- | --- | --- | --- |
| I | Wu CW | 2012 | miR-21 | N | - | Absolute | Y | RNeasy Mini Kit (Qiagen) |
| II | Wu CW | 2012 | miR-92a | N | - | Absolute | Y | RNeasy Mini Kit (Qiagen) |
| III | Wu CW | 2012 | miR-21, miR-92a | Y | - | Absolute | Y | RNeasy Mini Kit (Qiagen) |
| IV | Shigeru K | 2013 | miR-21 | N | custom miRNA | Relative | Y | unknown |
| V | Shigeru K | 2013 | miR-92a | N | custom miRNA | Relative | Y | unknown |
| VI | Wu CW | 2014 | miR-135b | N | - | Absolute | Y | RNeasy Mini Kit (Qiagen) |
| VII | Liu H | 2016 | miR-21 | N | RUN6B | Relative | Y | TRIzol |
| VIII | Liu H | 2016 | miR-146a | N | RUN6B | Relative | Y | TRIzol |
| IX | Liu H | 2016 | miR-21, miR-146a | Y | RUN6B | Relative | Y | TRIzol |
| X | Wu CW | 2017 | miR-451a, miR-144-5p | Y | miR-200b-3p | Relative | Y | RNeasy Mini Kit (Qiagen) |

**Supplementary Figure 1
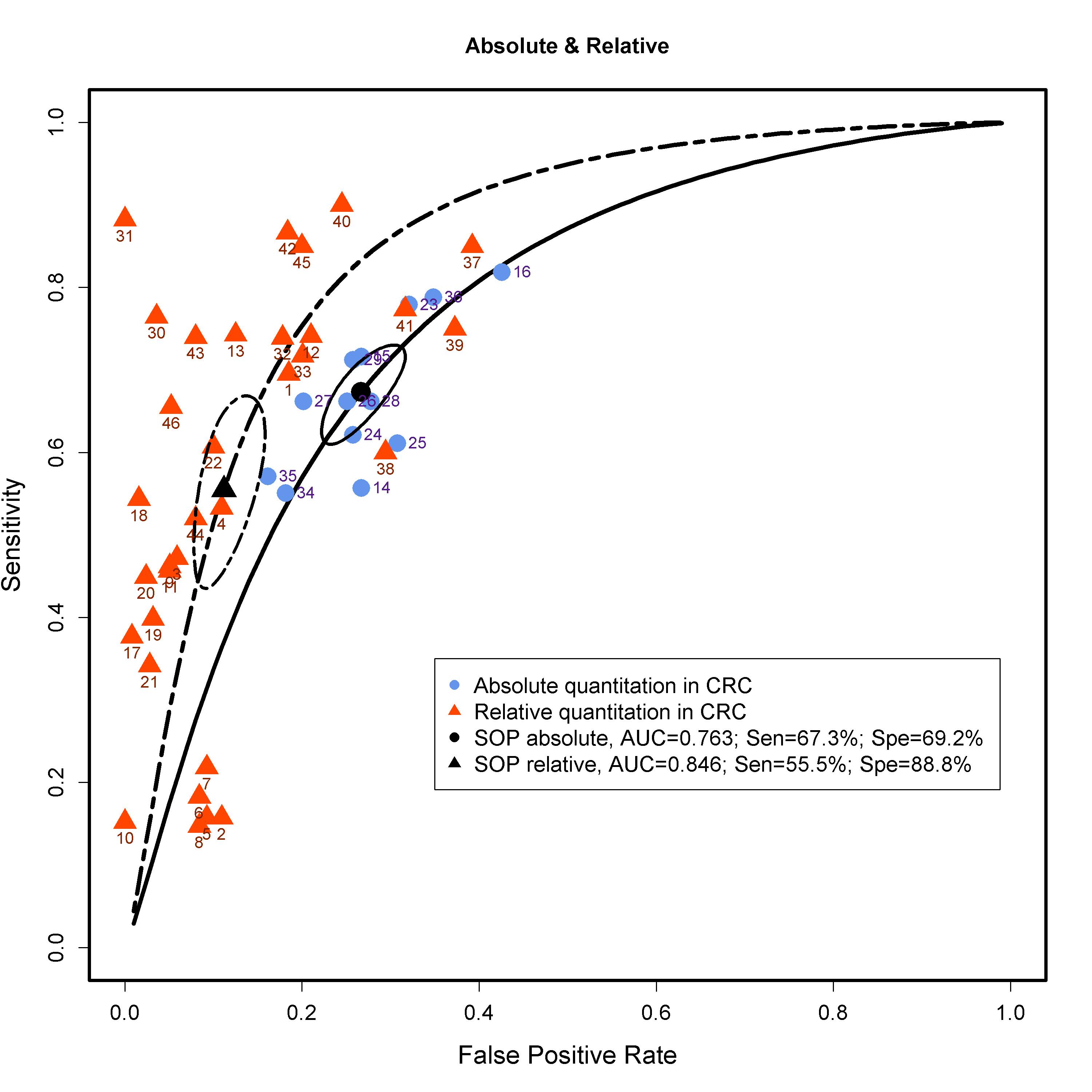
**

**Suppl. Fig. 1. Diagnostic accuracy in absolute versus relative quantification approach of colorectal cancer.** Summary receiver operating characteristic curves (SROC) for absolute (n = 13) and relative (n = 33) quantification approaches. The number next to the blue dot/ red triangle corresponds to the study ID in Table I. The circular regions (95% confidence contour) contain likely combinations of the mean value of sensitivity and specificity. Sen, sensitivity; Spe, specificity; SOP, summary operating point. # Absolute quantification: based on standard curves plotted by known input among all of the miRNAs and normalised to per nanogram of the total input RNA; Relative quantification: the target miRNA(s) normalised by a housekeeping small RNA.

**Supplementary Figure 2**


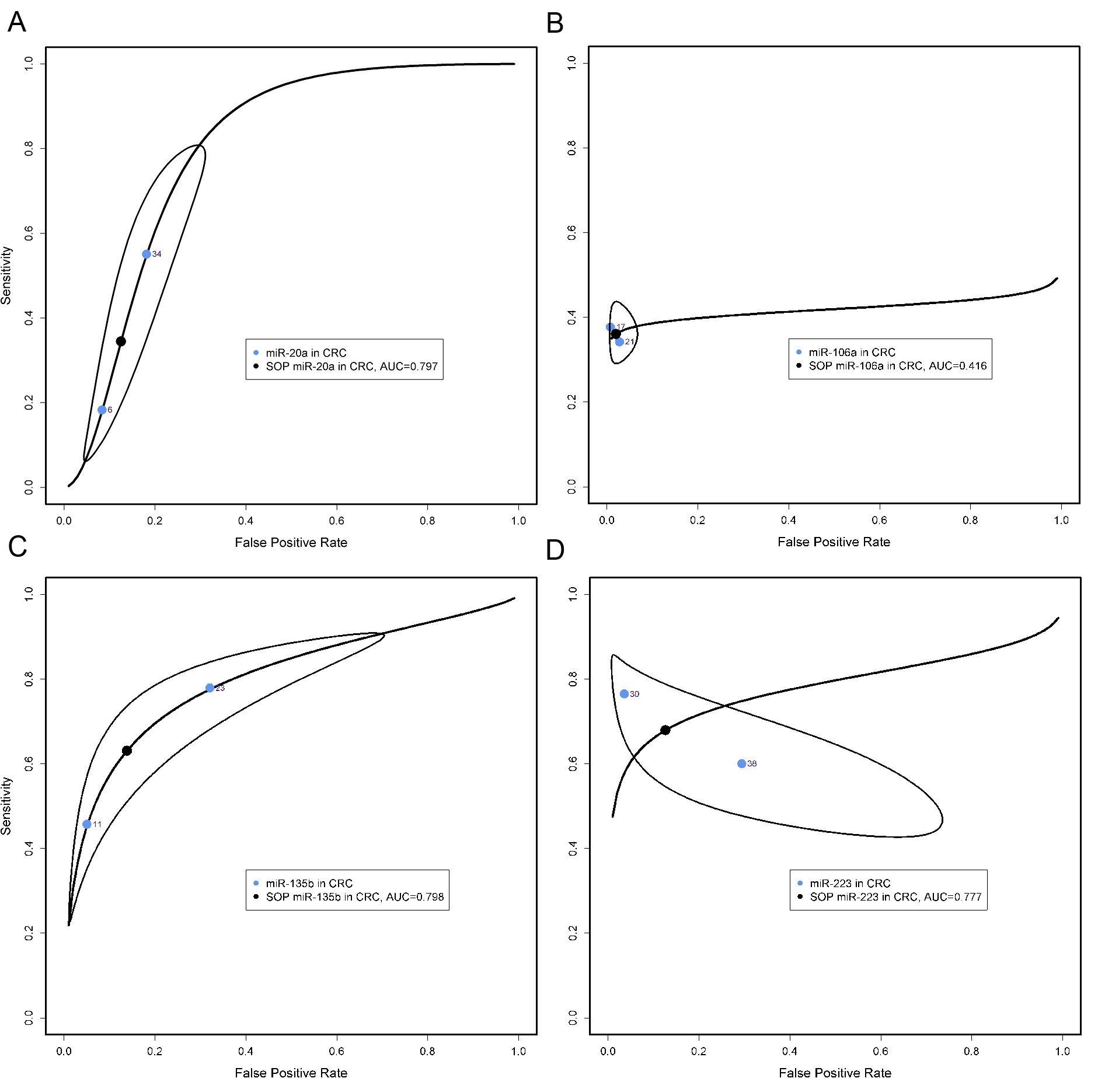


**Suppl. Fig. 2. Diagnostic accuracy in pooled miR-20a, miR-106a, miR-135b and miR-223.** All the Summary receiver operating characteristic curves for pooled (**A**) miR-20a, (**B**) miR-106a, (**C**) miR-135b and (**D**) miR-223 in the detection of CRC (n = 2). The number next to the blue dot corresponds to the study ID in Table I. The circular regions (95% confidence contour) contain likely combinations of the mean value of sensitivity and specificity. Sen, sensitivity; Spe, specificity; SOP, summary operating point.

**Supplementary Figure 3
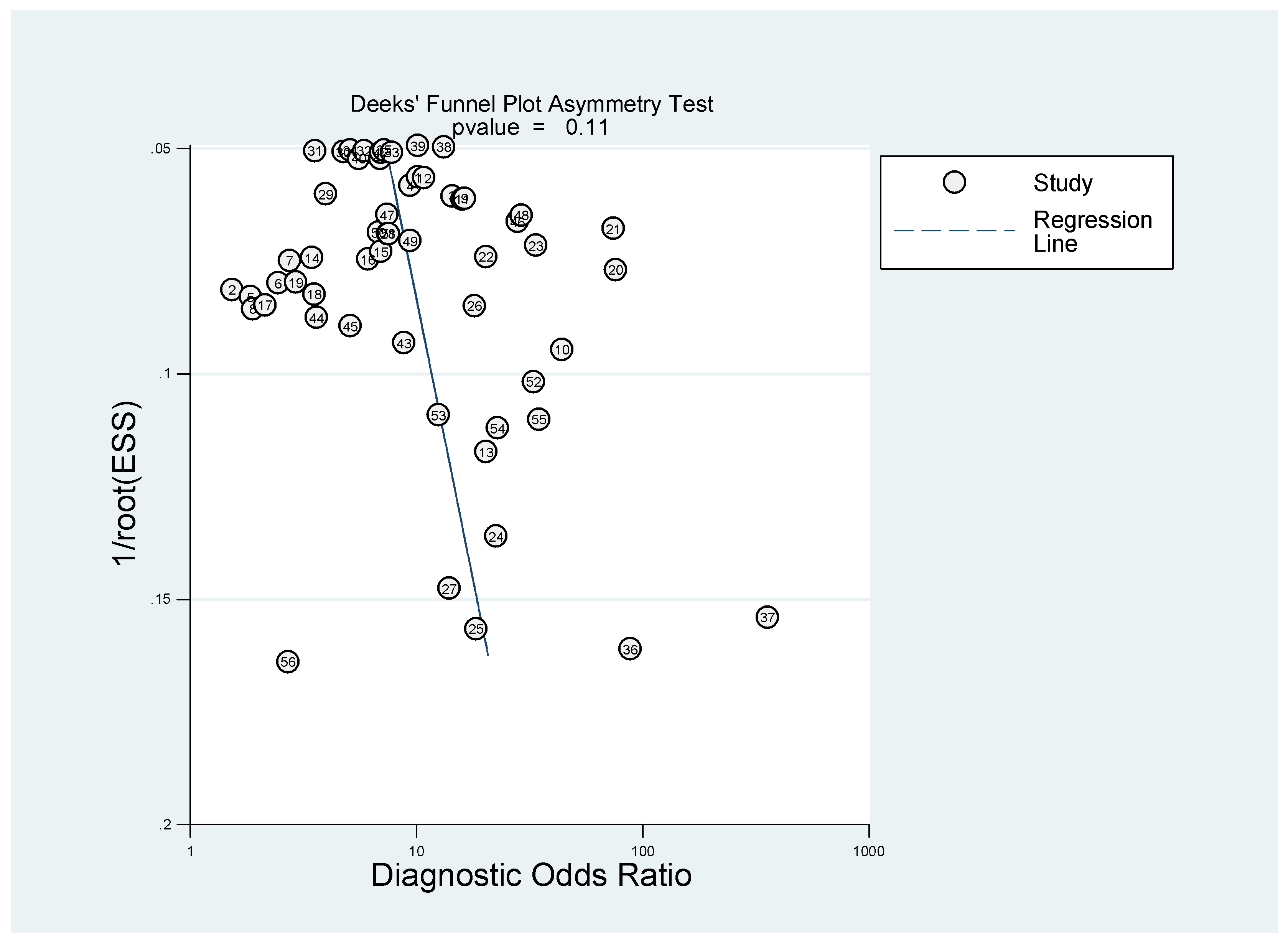
**

**Suppl. Fig. 3**. **Deeks’ funnel plot asymmetry test.** Test used for the assessment of potential bias in overall microRNA assays.

**Supplementary Figure 4
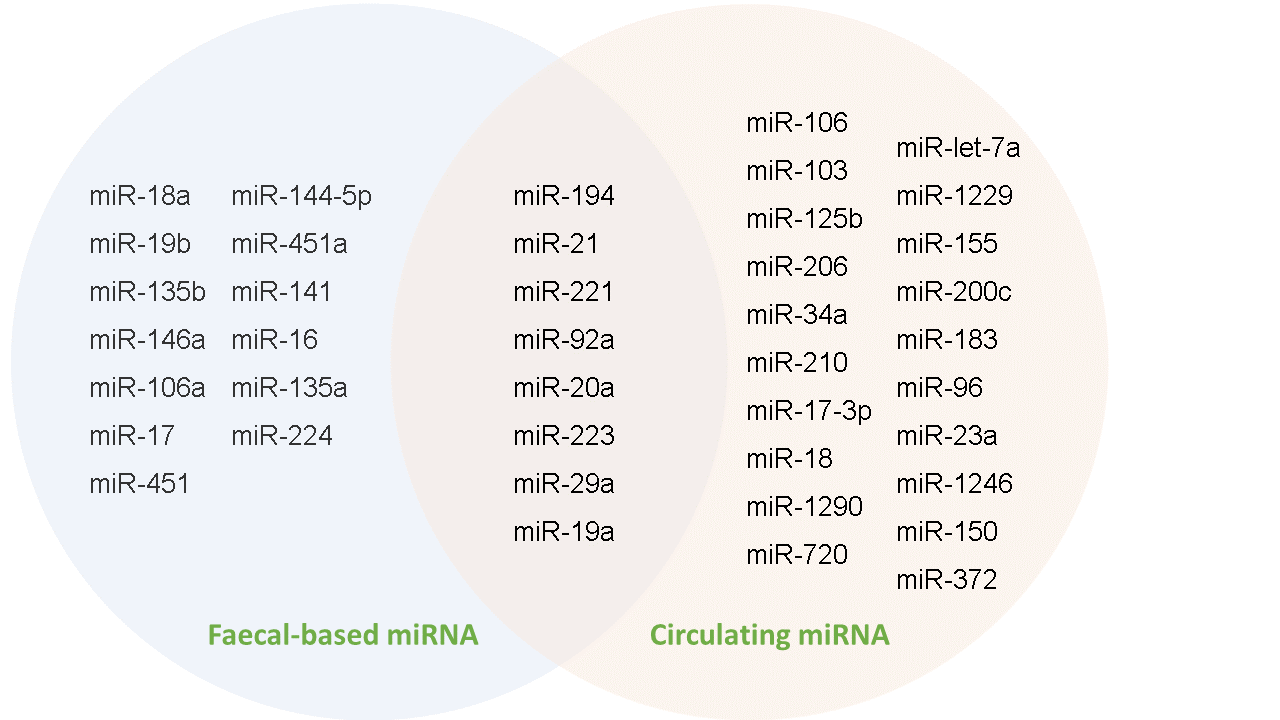
**

**Suppl. Fig. 4. Comparison of faecal-based and circulating miRNA for CRC screening.** A list of up-regulated circulating miRNAs (n = 28) retrieved from Taiyoma et al. (2018)^1^ was compared to our findings (n = 21). Among them, eight miRNAs are detected in both faecal and liquid biopsy samples.

**REFERENCE**

1. Toiyama, Y., Okugawa, Y., Fleshman, J., Richard Boland, C. & Goel, A. MicroRNAs as potential liquid biopsy biomarkers in colorectal cancer: A systematic review. *Biochim. Biophys. acta. Rev. cancer* **1870**, 274–282 (2018).

**Supplementary data 1**

|  | 14/04/16 | 05/01/17 | 17/11/17 |
| --- | --- | --- | --- |
| PubMed | 75 | 81 | 88 |
| Ovid Embase 1974 to 2017 Nov 16 | 117 | 142 | 247 |
| The Cochrane Library | 1 | 1 | 1 |
| Scopus | 103 | 119 | 144 |
| Web of Science (all databases) | 140 | 167 | 191 |
| TOTAL | **436** | **510** | **671** |
| DEDUPLICATED TOTAL |  | 230 | 318 |
| TOTAL – NEW SINCE 14/04/16 |  | 38 |  |
| Unique since 05/01/17 |  |  | 93 |

## PubMed:

((((("Feces"[Mesh]) OR ((fecal*[Title/Abstract] OR faecal*[Title/Abstract] OR feces*[Title/Abstract] OR faeces*[Title/Abstract] OR stool*[Title/Abstract])))) AND (("MicroRNAs"[Mesh]) OR (("micro RNA*"[Title/Abstract] OR microRNA*[Title/Abstract] OR miRNA*[Title/Abstract]))))) AND ((((((colorect*[Title/Abstract] OR colon[Title/Abstract] OR colonic[Title/Abstract] OR rect*[Title/Abstract] OR anal*[Title/Abstract] OR anus*[Title/Abstract] OR intestin*[Title/Abstract] OR bowel*[Title/Abstract]))) AND ((carcinom*[Title/Abstract] OR neoplas*[Title/Abstract] OR adenocarcinom*[Title/Abstract] OR cancer*[Title/Abstract] OR tumor*[Title/Abstract] OR tumour*[Title/Abstract] OR sarcom*[Title/Abstract] OR metastas*[Title/Abstract] OR malignan*[Title/Abstract] OR adenoma*[Title/Abstract])))) OR "Colorectal Neoplasms"[Mesh])

Otherwise:

1.       Colorectal Neoplasms/

2.       (colorect* or colon or colonic or rect* or anal* or anus* or intestin* or bowel*).ti,ab.

3.       (carcinom* or neoplas* or adenocarcinom* or cancer* or tumor* or tumour* or sarcom* or metastas* or malignan* or adenoma*).ti,ab.

4.       2 AND 3

5. 1 OR 4

6.       Feces/

7.       (fecal* OR faecal* OR feces* OR faeces* OR stool*).ti,ab.

8.       6 OR 7

9.       MicroRNAs/

10.       (“micro RNA*” OR microRNA* OR miRNA*).ti,ab.

11.   9 OR 10

12.   8 AND 11

13.   5 AND 12

## Embase 1974 to 2017 November 16

1. exp colorectal tumor/
2. exp colon cancer/
3. exp rectum cancer/
4. (colorect* or colon or colonic or rect* or anal* or anus* or intestin* or bowel*).ti,ab.
5. (carcinom* or neoplas* or adenocarcinom* or cancer* or tumor* or tumour* or sarcom* or metastas* or malignan* or adenoma*).ti,ab.
6. 4 and 5
7. 1 or 2 or 3 or 6
8. exp feces/
9. (fecal* or faecal* or feces* or faeces* or stool*).ti,ab.
10. 8 or 9
11. exp microRNA/
12. ("micro RNA*" or microRNA* or miRNA*).ti,ab.
13. 11 or 12
14. 10 and 13
15. 7 and 14

## SCOPUS

( TITLE-ABS KEY ( colorect*  OR  colon  OR  colonic  OR  rect*  OR  anal*  OR  anus*  OR  intestin*  OR  bowel* ) )  AND  ( TITLE-ABS-KEY ( carcinom*  OR  neoplas*  OR  adenocarcinom*  OR  cancer*  OR  tumor*  OR  tumour*  OR  sarcom*  OR  metastas*  OR  malignan*  OR  adenoma* ) )  AND  ( TITLE-ABS-KEY ( fecal*  OR  faecal*  OR  feces*  OR  faeces*  OR  stool* ) )  AND  ( TITLE-ABS-KEY ( "micro RNA*"  OR  microrna*  OR  mirna* ) )

## Web of Science – ALL DATABASES

1. TOPIC: (colorect* or colon or colonic or rect* or anal* or anus* or intestin* or bowel*) OR TITLE: colorect* or colon or colonic or rect* or anal* or anus* or intestin* or bowel*

2. TOPIC: (carcinom* or neoplas* or adenocarcinom* or cancer* or tumor* or tumour* or sarcom* or metastas* or malignan* or adenoma*) OR TITLE: (carcinom* or neoplas* or adenocarcinom* or cancer* or tumor* or tumour* or sarcom* or metastas* or malignan* or adenoma*)

3. TOPIC: (fecal* or faecal* or feces* or faeces* or stool*) OR TITLE: (fecal* or faecal* or feces* or faeces* or stool*)

4. TOPIC: ("micro RNA*"  OR  microrna*  OR  mirna*) OR TITLE: ("micro RNA*"  OR  microrna*  OR  mirna*)

5. 1 AND 2 AND 3 AND 4

## The Cochrane Library

Date Run: 17/11/17

ID Search Hits

#1 MeSH descriptor: [Colorectal Neoplasms] explode all trees 6544

#2 (colorect* or colon or colonic or rect* or anal* or anus* or intestin* or bowel*) .ti,ab. 3372

#3 (carcinom* or neoplas* or adenocarcinom* or cancer* or tumor* or tumour* or sarcom* or metastas* or malignan* or adenoma*) .ti,ab. 1787

#4 #2 and #3 1787

#5 #1 or #4 8309

#6 MeSH descriptor: [Feces] explode all trees 2591

#7 (fecal* or faecal* or feces* or faeces* or stool*) .ti,ab. 256

#8 #6 or #7 2847

#9 MeSH descriptor: [MicroRNAs] explode all trees 193

#10 ("micro RNA*" or microRNA* or miRNA*) .ti,ab. 8

#11 #9 or #10 201

#12 #8 and #11 1

#13 #5 and #12 1
